# Supplementary material for: A QbD Approach to Design and to Optimize the Self-Emulsifying Resveratrol–Phospholipid Complex to Enhance Drug Bioavailability through Lymphatic Transport
Source: Polymers (Basel). 2022 Aug 8;14(15):3220. doi: 10.3390/polym14153220 (PMC9371077; doi:10.3390/polym14153220)
Supplement: Supplementary file 1 [file polymers-14-03220-s001.zip › polymers-1783144-supplementary.pdf]

Supplementary materials

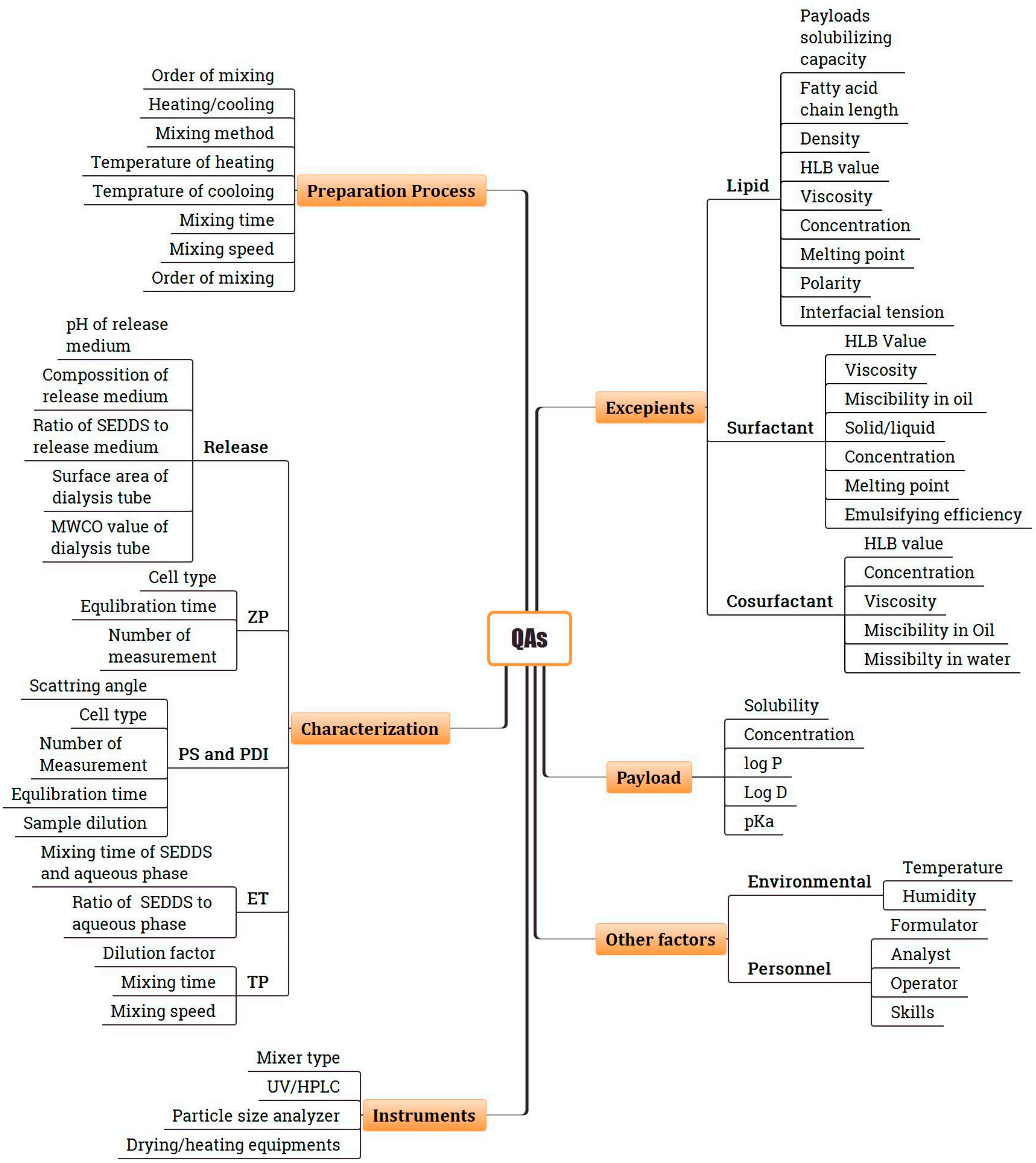

Figure S1. Diagram of factors affecting QAs.

Table S1. Emulsification time and transmittance percentage of different concentrations of oil, surfactant, and co-surfactant, examined in 11 different points

Points	Emulsification time	% Transmittance
1	6 minutes 35 seconds	96.350
2	2 minutes 46 seconds	95.670
3	1 minute 14 seconds	95.642
4	2 minute 8 seconds	94.343
5	1 minute 3 seconds	93.140
6	46 seconds	89.160
7	2 minutes	96.509
8	35 seconds	90.050
9	15 seconds	67.674
10	7 minutes 32 seconds	85.120
11	4 minutes 38 seconds	79.850

Table S2. Emulsification time and transmittance percentage data of different concentrations of TPGS were examined at 7 points.

	5% TPGS		10% TPGS	
Points	**ET**	**TP**	**ET**	**TP**
2	2 min 33 seconds	88.456	38 seconds	92.281
3	1 min 11 seconds	86.312	38 seconds	95.014
4	Not dispersed	84.505	Not dispersed	94.372
5	1 min 10 seconds	88.131	42 seconds	93.103
6	40 seconds	89.217	24 seconds	91.176
7	Not dispersed	89.148	Not dispersed	96.513
8	2 min 45 seconds	88.383	47 seconds	90.046

Table S3. The polynomial model fitting summary of CQA responses.

**CQA responses**	**The best-fitted polynomial model**	**Fit summery**					
		**Sequential p-value**	**R^2**	**Adjusted R^2**	**Predicted R^2**	**Adjusted R^2 to Predicted R^2 difference**	**Adequate Precision**
$\mathbf{Y_{ET}}$	Cubic	0.0006	0.9991	0.9965	0.9686	0.0279	22.83
$\mathbf{Y_{GS}}$	Sp. Quartic vs. Quadratic	0.0345	0.9870	0.9609	0.8721	0.0888	26.43
$\mathbf{Y_{PDI}}$	Cubic	0.0056	0.9931	0.9723	0.9024	0.0699	67.9
$\mathbf{Y_{\mathit{Release}}}$	Cubic	0.0005	0.9987	0..9946	0.9815	0.0131	59.53

Table S4. The optimization criteria of CMAs and CQAs responses.

CMAs	Notation	Goal	Limit range	Importance
Labrafil® M 1944 CS concentration	X_1	Maximize	0.1 – 0.3	+++++
Kolliphor® RH 40 concentration	X_2	In range	0.4 – 0.6	+++
Transcutol® HP concentration	X_3	Minimize	0.3 – 0.4	+++++
**CQAs responses**				
Globule size (GS)	Y_{GS}	In range	10 – 30 nm	+++
Polydispersity index (PDI)	Y_{PDI}	Minimize	0 – 0.2	+++
Emulsification time (ET)	Y_{ET}	In range	1 – 60 sec	+++
Release	$Y_{Release}$	In range	80 – 100%	+++

Table S5. The verification points were shown as the weight ratio of oil, surfactant, and co-surfactant. Alongside, the CQA responses of the verification runs with their predicted mean and 95% prediction intervals were also shown.

Weight ratio of Labrafil® 1944 CS, Kolliphor® RH40, and Transcutol® HP	CQA response	Predicted mean	Actual	95% PI low	95% PI high
**VR1** Ratio (0.225029, 0.415328, 0.359643)	Y_{ET}	23.117	21.67	15.4463	30.7877
	Y_{GS}	21.3637	22.07	18.5315	24.196
	Y_{PDI}	0.162309	0.148	0.130284	0.194334
	$Y_{Release}$	82.9224	83.93	80.8606	84.9842
**VR2** Ratio (0.1875, 0.4375, 0.375)	Y_{ET}	23.941	22.63	14.6357	33.2463
	Y_{GS}	18.9608	21.88	15.3844	22.5371
	Y_{PDI}	0.124196	0.097	0.0837577	0.164634
	$Y_{Release}$	92.4581	90.28	89.8546	95.0616
**VR3** Ratio (0.117018, 0.512281, 0.370701)	Y_{ET}	30.697	28.97	21.3968	39.9973
	Y_{GS}	17.5576	20.03	13.9491	21.1661
	Y_{PDI}	0.191211	0.148	0.150409	0.232013
	$Y_{Release}$	91.9037	93.58	89.2768	94.5306
**VR4** Ratio (0.167778, 0.437319, 0.394903)	Y_{ET}	23.6608	26.68	14.1414	33.1802
	Y_{GS}	21.5204	23.57	18.2895	24.7512
	Y_{PDI}	0.183661	0.196	0.147129	0.220193
	$Y_{Release}$	92.1088	90.17	89.7568	94.4608

**VR5** Ratio (0.2375, 0.4375, 0.325)	Y_{ET}	29.3715	34.78	20.0466	38.6965
	Y_{GS}	35.1401	32.47	30.4895	39.7908
	Y_{PDI}	0.482679	0.416	0.430094	0.535265
	$Y_{Release}$	74.013	76.19	70.6274	77.3985
**VR6** Ratio (0.1375, 0.5375, 0.325)	Y_{ET}	55.439	58.17	47.7479	63.1301
	Y_{GS}	29.5001	29.02	25.2521	33.7482
	Y_{PDI}	0.50567	0.487	0.457636	0.553704
	$Y_{Release}$	74.5334	76.18	71.441	77.6259
**VR7** Ratio (0.1375, 0.4875, 0.375)	Y_{ET}	34.4499	36.01	26.7826	42.1172
	Y_{GS}	16.8158	18.86	13.7813	19.8502
	Y_{PDI}	0.131284	0.158	0.096973	0.165595
	$Y_{Release}$	93.5607	90.29	91.3517	95.7697
**VR8** Ratio (0.16655, 0.466395, 0.367055)	Y_{ET}	33.1813	37.67	26.6106	39.752
	Y_{GS}	19.664	21.24	16.9449	22.3832
	Y_{PDI}	0.170305	0.196	0.139559	0.201051
	$Y_{Release}$	91.6925	93.09	89.713	93.6719
